# Environmental factors in autoimmune diseases and their role in multiple sclerosis

**DOI:** 10.1007/s00018-016-2311-1

**Published:** 2016-08-04

**Authors:** Stefanie Jörg, Diana A. Grohme, Melanie Erzler, Marilene Binsfeld, Aiden Haghikia, Dominik N. Müller, Ralf A. Linker, Markus Kleinewietfeld

**Affiliations:** 1 University Hospital Erlangen at the Friedrich-Alexander-University (FAU) Erlangen-Nuremberg, Erlangen, Germany; 2 Translational Immunology, Department of Clinical Pathobiochemistry, Medical Faculty Carl Gustav Carus, TU Dresden, Dresden, Germany; 3 Center for Regenerative Therapies Dresden (CRTD), Dresden, Germany; 4 VIB Laboratory of Translational Immunomodulation & Hasselt University, Diepenbeek, Belgium; 5 Department of Neurology, Ruhr-University Bochum, Bochum, Germany; 6 Experimental and Clinical Research Center, An Institutional Cooperation Between the Charité Medical Faculty and the Max-Delbruck Center for Molecular Medicine, Berlin, Germany

**Keywords:** Autoimmunity, MS, CD4^+^ T cells, Foxp3^+^ Tregs, Th17 cells, Environmental risk factors, Microbiota

## Abstract

An increase in autoimmune diseases poses a socioeconomic challenge worldwide. Predisposing genetic risk has been identified, yet environmental factors make up a significant part of the risk in disease initiation and propagation. Next to improved hygiene and a gross reduction of infections, changes in dietary habits are one of the most evident *Western lifestyle* factors potentially associated with the increase in autoimmune diseases. Growing evidence suggests that particularly a typical ‘Western diet’, rich in saturated fat and salt and related pathologies can have a profound impact on local and systemic immune responses under physiologic and autoimmune conditions such as in multiple sclerosis (MS). In this review, we discuss recent findings on environmental factors influencing autoimmunity with an emphasis on the impact of ‘Western diet’ on immune homeostasis and gut microbiota in MS.

## Introduction

The common cause for autoimmune diseases, such as multiple sclerosis (MS), type-1 diabetes (T1D), rheumatoid arthritis (RA) or inflammatory bowel disease (IBD), is suspected to be the loss of tolerance to self. Initiating events are mostly unknown but may be associated with a broad range of overstimulated and mislead immune responses, including loss of costimulatory control by antigen presenting cells (APC) and “molecular mimicry”, which causes immune cells recognizing a certain extrinsic antigen to react also to structurally similar self-antigens [1]. Once an immune reaction is initiated, massive invasion of immune cells accompanied by a systemic/humoral immune response leads to tissue damage resulting in exposure of additional self-antigens [1, 2]. Although predisposing genetic risk factors have been identified for various autoimmune diseases, it is understood that they account only for a fraction of the overall disease [3, 4]. Hence, the remaining risk component of autoimmune diseases such as MS must be related to exogenous factors. Research efforts of the past decades confirm this finding, and it is now well accepted that the etiology of many autoimmune diseases involves environmental factors that act on top of genetic susceptibility profiles  [3, 5, 6]. The increasing incidence of autoimmune diseases with a high prevalence in Western countries [7, 8] and the rapid evolution of MS in former low prevalence countries like Japan [9] nurture multiple explanatory concepts around environmental triggers. The so-called hygiene hypothesis aims to explain the increase in autoimmunity in industrialized countries by linking the decrease of infection rates and the increase in autoimmune diseases to a general improvement of hygiene standards [10].

Besides infection, there are many more environmental factors that have been proposed to promote autoimmune diseases, like MS, including climate, stress, occupation, cigarette smoking, and diet [11]. Of note, the consumption of ‘Westernized food’, including high salt, high fat, high protein, and high sugar intake, has already been associated with increasing prevalence in various diseases [12, 13]. The change of dietary habits has been under intensive investigation, revealing a direct influence on immune homeostasis and on bacterial communities colonizing the gastrointestinal tract (GIT) [14] and the gut microbiota is tightly connected to the immune system and highly involved in immune regulatory processes [15]. IBD has been associated with shifts and variety reduction in the microbiome. This observation has also been made in other autoimmune diseases not directly associated with the GIT [12, 16]. However, there is still little understanding on the mechanisms linking environmental factors to disease mechanisms, genetic predisposition, and the immune system. Gaining further insight into the influence of environment and microbiota on immune homeostasis will be a powerful source for a better understanding of the rising incidence of autoimmune pathologies with the aim to provide novel approaches for therapeutic treatment and prevention strategies.

This review will, therefore, discuss recent developments in research linking the environment to autoimmune diseases with an emphasis on the nexus of immune cells, dietary components, and gut microbiota. Thereby, we highlight the role of CD4^+^ T lymphocytes in MS, especially with respect to the importance of balancing effector and regulatory T cells for maintenance of immune homeostasis.

## Immune cells in multiple sclerosis

Worldwide, there are an estimated 2.5 million patients suffering from MS with women twice as frequently affected as men [17]. MS is a progressive demyelinating disease characterized by disseminated central nervous system (CNS) lesions, most likely caused by an autoimmune response to CNS self-antigens [18]. Pathologically, perivascular inflammatory infiltrates in brain, optic nerve, and spinal cord dominate during the early phases of the disease. These infiltrates contain mononuclear immune cells, such as lymphocytes (CD4^+^ and CD8^+^ T cells as well as B cells), monocytes, and macrophages, and form so-called plaques, the end stage of inflammation, characterized by demyelination, astrogliosis, and neuronal as well as axonal degeneration  [5, 19, 20]. Dendritic cells (DCs) have been shown to play a critical role in immune invasion of the CNS by presenting antigen to activated autoreactive T cells [21, 22]. In addition, the activation of microglia and macrophages plays an essential role in the pathogenesis of the disease [23]. Recent studies, demonstrating the presence of inflammatory cells and their products in CNS lesions, led to the generally accepted hypothesis that at least relapsing-remitting MS is triggered by pathogenic CD4^+^ T cells reactive against myelin constituents [5, 19, 20]. Besides these, several other cells of the innate and adaptive immune system are involved in the pathogenesis of MS. For instance, CD8^+^ T cells were shown to directly damage axons by the secretion of granzyme B and perforin,  [24] and macrophages can contribute to tissue damage by releasing toxic molecules like nitric oxide, oxygen radicals and proinflammatory cytokines [25]. Also B cells [26–28], as well as innate lymphoid cells (ILCs) [29], γ/δ T cells [30] and NK cells [31] play distinct important roles in the autoimmune response. In particular, the recently discovered ILCs, which are tissue-resident lymphoid cells that lack specific antigen receptors, gained interest as new targets for modulating immune tolerance in autoimmune diseases like MS [29]. ILCs have been recognized for their importance in mediating the interplay between microbiota and the immune system [32], and the non-cytotoxic ILC1, ILC2, and ILC3 show a striking resemblance to CD4^+^ T cell subsets with respect to development and function [33, 34].

Many data on the immunopathology of MS stem from experimental autoimmune encephalomyelitis (EAE), an animal model mimicking several aspects of the disease [35–37]. EAE is most commonly induced in rodents by the immunization with myelin peptides (e.g., MOG, myelin oligodendrocyte glycoprotein) and adjuvant [38] or the transfer of myelin-reactive T cells [39]. The resulting T cell-mediated acute autoimmune reaction against myelin in the CNS induces similar symptoms to those seen in MS. It was thus initially shown in EAE models that CD4^+^ T helper (Th) cells play a key role in MS. MS was first thought to be a Th1 cell-mediated autoimmune disease, with interferon gamma (IFN-γ) assuming a pathogenic role, while Th2 cells producing primarily interleukin (IL)-4 or IL-10 exert a modulatory function with a protective role  [40]. After the identification of the relevance of IL-23 in EAE, subsequent work showed that also Th17 cells are involved in the pathogenesis of the disease, since IL-23 is a critical growth factor for this cell subset [41, 42].

Th17 cells frequently occur in the intestine, playing a particularly important role in the intestinal immune homeostasis by providing defense against extracellular bacteria and clearance of pathogens  [43]. Depending on their physiological role, Th17 cells are exposed to environmental factors and could, therefore, be influenced by nutritional components. Together with Th1 cells, Th17 cells are believed to be the main constituents of the CD4^+^ T effector subset that drives disease pathology in T cell-dependent autoimmune diseases [44]. However, due to the fact that Th17 cells are associated with several autoimmune diseases, recent investigations often focus on this Th cell subset  [44]. In EAE studies, IL-17 deficient mice showed delayed and reduced symptoms, but no complete protection  [45, 46]. More recent studies found that the pathogenicity of Th17 cells additionally depends on the IL-23-induced production of the cytokine granulocyte macrophage colony-stimulating factor (GM-CSF), probably explaining the incomplete protection from EAE in IL-17-deficient mice [47, 48]. The importance of Th17 cells was also linked to MS, as lesions of MS patients contained an increased frequency of IL-17-producing CD4^+^ T cells [49]. Moreover, it was found that Th17 cells are able to cross the inflamed blood–brain barrier and secrete the proinflammatory cytokine IL-17A [50]. A recent study showed that in particular Th17 instead of Th1 responses were absent in patients with MS disease abrogation after hematopoietic stem cell transplantation [51], confirming the pivotal role of Th17 cells in MS.

In contrast to effector T cells, regulatory T cells (Tregs) play a central role in immunoregulatory reactions and suppression of autoreactive immune cells. Once activated, forkhead box P3 (FoxP3) positive Tregs exert their suppressive functions via the release of anti-inflammatory cytokines like IL-10 and transforming growth factor (TGF)-β in addition to cell–cell contact-dependent mechanisms [52]. In EAE, adoptive transfer of Tregs improved disease symptoms, while ablation led to worsening of disease [52]. Importantly, an impairment of regulatory T cell function is frequently observed in patients with autoimmune diseases like MS, and it is believed to be a major cause for disruption of immune homeostasis, further contributing to autoimmune reactivity [2, 52]. The loss of Treg suppressive capacity might be related to the potential of Tregs to convert into Th1-like Tregs, secreting IFN-γ [53–55], as well as Th17-like Tregs, secreting IL-17 with a proinflammatory potential [56–59]. In MS patients, IFN-γ-secreting Tregs were found to be increased [60] and Tregs displayed lower expression levels of FoxP3 and an impaired suppressive capacity [2, 52, 61–63]. It is thus well accepted that the balance between T effector cells and Treg subsets plays a major role in autoimmune diseases like MS.

## Environment and its link to MS

Genetic variants influencing susceptibility or protection from autoimmune diseases like MS have been explored in studies of twin concordance [64], familiar clustering and genome wide associations studies (GWAS) [65]. Genes account for roughly 25–30 % inheritability in monozygotic twins, and as in the majority of autoimmune diseases, variants of the human leukocyte antigen (HLA) complex provide a strong susceptibility for MS. Especially, the HLA-DRB1*1501 allele is associated with a highly elevated risk for MS development, displaying a sixfold risk increase in homozygous carriers [65]. In addition, a number of immune-related risk-alleles have been established independently of the HLA-locus, affecting in particular CD4^+^ T cell subset responses [66, 67]. Among the identified genes are, for instance, the IL-2 and IL-7 receptors, both genes playing a critical role for Tregs and Th effector cells [66, 67]. Moreover, predisposing variants for MS have been found for the Th17/IL-23 axis, supporting a role for Th17 cells in disease development [66]. Similar studies have also suggested an increased susceptibility caused by variants of CD86, an important coreceptor for T cell stimulation expressed by DCs, supporting the important role of DC/T cell interaction for MS [65]. A genetic link to B cell function and MS is for instance based on risk variants in the *CD40* and *CXCR5* genes, encoding for a surface protein inducing B cell activation and differentiation and a chemokine receptor expressed on B and T cells [28]. GWAS studies have even identified risk association with genes that are important for current and new MS therapies including vascular cell adhesion molecule 1 (VCAM1), as well as genes related to the crucial environmental factor vitamin D [65]. Nevertheless, the large amount of data on genetic predisposition for autoimmune diseases can, in most of the cases, only explain a part of the disease risk, supporting the view that the increasing prevalence of MS is triggered in addition by environmental factors. Epidemiological studies have shown a striking trend of MS toward higher prevalence with increasing latitude and an increase in disease incidence in developed countries [7, 8]. Several theories have aimed to explain these observations. Since exposure to sunlight is the main source for vitamin D synthesis in humans, a lower sunlight exposure in high-risk regions such as Northern Europe results in a decreased generation of vitamin D. In fact, patients with MS were found to have lower blood levels of vitamin D [68, 69] and also in other autoimmune diseases, low vitamin D levels may represent an emerging risk factor [70, 71]. Studies on the effect of vitamin D in autoimmunity suggest an immunomodulatory capacity with anti-inflammatory action [72, 73]. Moreover, UVB light increased levels of tolerogenic DCs and Tregs while reducing effector T cell counts in MS patients [74]. In addition to sunlight, fatty fish could be a dietary source of vitamin D, and fish consumption correlated with lower MS prevalence in coastal areas [75]. However, the recent OFAMS study failed to detect any influence of fish oil on the course of MS [76]. Nevertheless, there is an increasing amount of data demonstrating the positive role of vitamin D or other vitamins like biotin [77] in MS, as summarized elsewhere in depth [78–80].

Furthermore, the hygiene hypothesis states that individuals not exposed to certain infections early in life but growing up with improved hygiene may develop a hyperalert immune system, favoring the occurrence of autoimmune diseases [10, 81]. While according to the hygiene hypothesis some infectious agents may be protective, others may increase the risk, such as Epstein–Barr virus (EBV) which is associated with MS especially when infection takes place in late adolescence [5]. A potential explanation for that might be the similarity of the EBV nuclear antigen (EBNA)-1 to myelin surface proteins, leading to crossreactivity of adaptive immune cells [82]. Interestingly, T cells specific to EBNA-1 display a significantly higher frequency as well as a broader specificity in MS patients than in healthy controls [83]. Although associations between MS and EBV infection have been well investigated, the role of EBV in MS pathology remains unclear as further studies could not demonstrate latent or active EBV infection in active MS lesions [84]. Infections that might confer a protective effect to the host are colonizations of the GIT by parasitic worms. Different studies have shown that helminths may affect the host’s immune response by the promotion of an anti-inflammatory environment [85, 86]. Based on this, the possibility of treating MS with helminths has already been explored in animal models [87–89] and also phase 1 clinical trials [90]. In line with these findings, the fact that colonization of the GIT by parasitic worms has decreased over the past decades in industrialized countries [91] possibly contributes to an increasing MS prevalence.

The gut and its microbiota recently gained a lot of attention in various fields of research. The human gut microbiome is expected to consist of more than 10^14^ bacterial cells from about 500–1000 species and helps the host to maintain the body in homeostasis [92]. The first exposure to the human microbiome occurs during birth, and breast milk or formula feeding further influences the colonization of the new born’s own gut microbiome [93–95]. During a lifetime, the human gut is recolonized permanently over the years with dominating bacterial orders, such as *Firmicutes* and *Bacteroidetes*, and other bacteria found in minor amounts, creating a unique human gut microbiota for each individual [16]. The human microbiota reaches maximum diversity at adolescence and can then be stable for years. A large amount of physiological functions, such as food digestion (providing fermentation products) and competition with potential pathogens, have been described for the commensal gut bacteria [96, 97]. Besides this, a vast collection of data points out that the gut microbiota is essential for the proper function of our immune system and metabolism and thus has a strong impact on human’s health. Indeed, changes in the gut microbiota have been observed in several diseases such as IBD [98], allergies [99], and asthma [100–102]. Those changes are induced by many factors, such as diet, stress or medication, and can lead to a so-called dysbiosis [103, 104]. A common consequence of dysbiosis is the alteration of the mucosal immune system leading to a rise of gut inflammation and alterations of intestinal immunity [105]. It was shown that a dysbiotic microbiome could lead to Treg deficiency and an activation of proinflammatory Th17 cells [106, 107]. While not expecting a direct correlation between the gut and brain autoimmunity, EAE studies indicate that the microbiota might play a role in MS as well. For instance, germ-free mice were shown to be protected from EAE induction, and using transgenic mice, Berer and colleagues could show that gut bacteria are a necessary prerequisite to induce a relapsing-remitting autoimmune disease [108]. Consistent with this, oral antibiotic treatment reduced and modulated bacterial populations in wild-type mice and, thereby, significantly ameliorated EAE onset and severity [109]. This effect was correlated with an increase in IL-10 producing Tregs, mainly induced by polysaccharide A of *Bacteroides fragilis* [110, 111]. Similar to Tregs, Th17 cells could be influenced by the gut microbiota. It was demonstrated that distinct species of commensal bacteria, namely, segmented filamentous bacteria (SFB), can specifically induce Th17 cells [112]. It was further shown that the induction of these cells could be indirectly influenced by luminal adenosine triphosphate (ATP) secreted from bacteria [113]. Moreover, the recently described gut–brain-axis may further support a possible link between gut microbiota and autoimmunity [114].

**Fig. 1:**
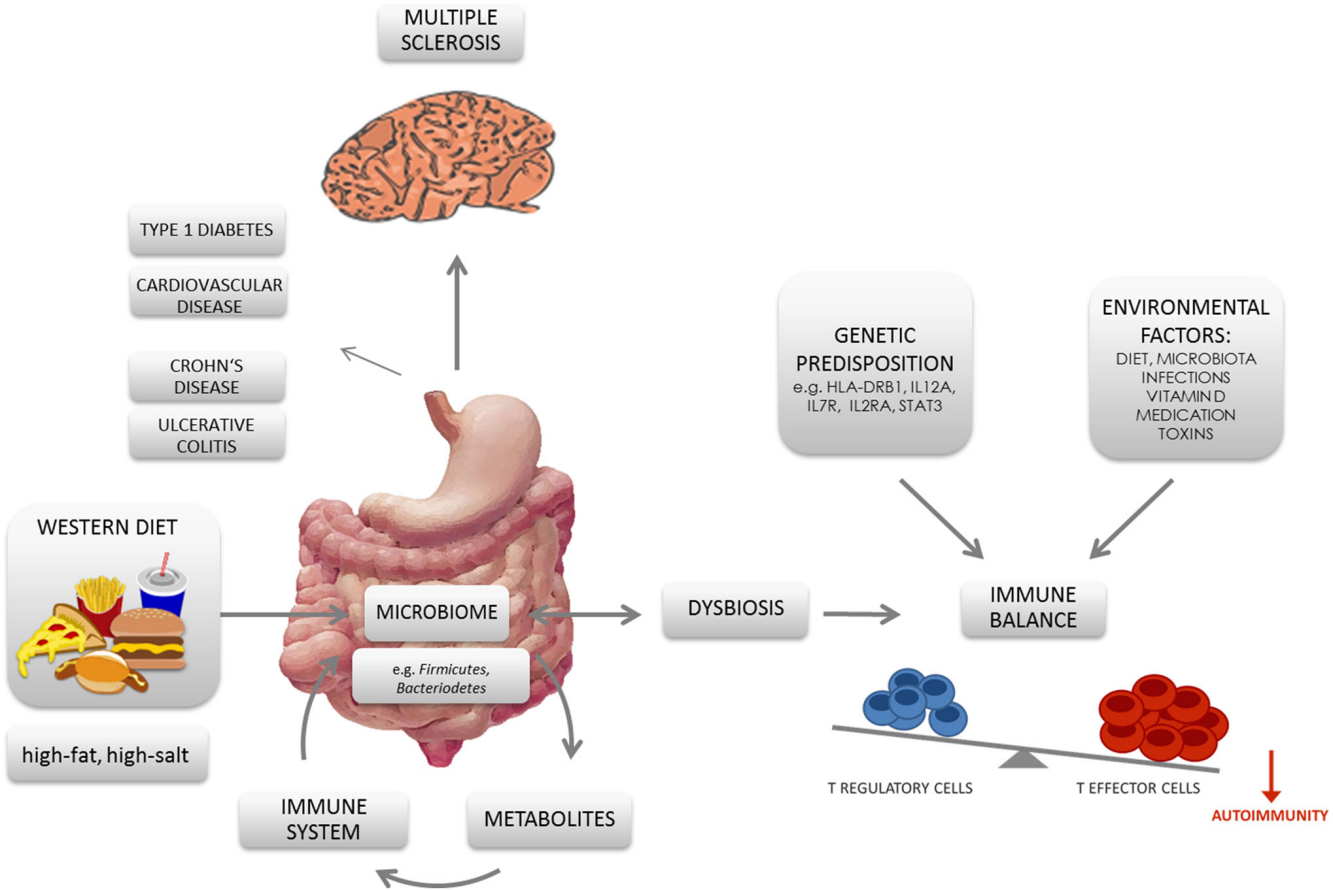
Schematic diagram displaying the nexus between ‘Western diet’, gut microbiota, T cells, and autoimmunity

In addition to direct effects of the microbiota, also dietary changes in association with a microbiota modulation are likely to be linked with the increasing incidences of autoimmune disorders [115]. It was shown that a ‘Western diet’ increases inflammation and might negatively affect the gut-immune homeostasis. In contrast, low-calorie diets based on fruits, fish and vegetables downregulate proinflammatory molecules and restore or maintain a healthy symbiotic gut microbiota [116]. For instance, plant-derived nutrients were found to be associated with an anti-inflammatory potential by acting as ligands of the aryl hydrocarbon receptor (AhR) [117, 118]. AhR acts as a transcription factor in a variety of immune cells, including Th17 and Tregs, and has been associated with susceptibility as well as prevention of autoimmune diseases depending on its ligands [107, 117, 118]. In that matter, indole-3-carbinol, deriving from crucifers such as broccoli, has been shown to suppress the production of proinflammatory cytokines [118, 119], whereas the tryptophan-derived AhR ligand FICZ (6-Formylindolo(3,2-b)carbazole) specifically increases the Th17 population and, therefore, worsens EAE severity [107]. Another plant-derived component supporting the induction of Tregs is retinoic acid (RA). RA is metabolized from vitamin A, which is derived from carotenoids in plants and as retinol from animals [118]. The highly metabolically active intermediate product all-trans RA (ATRA) can be generated by mucosal DCs potent in inducing and maintaining regulatory T cells [120, 121]. Nutrients, mostly discussed as risk factors in autoimmunity, are related to a ‘Western diet’. In that matter, especially, changes in the gut microbiome in association with diets rich in fat and salt have gained increasing attention during recent years and are discussed as potential risk factors for MS (Fig. 1).

## Fatty acids

Integral components of the daily diet that have recently been linked to autoimmunity via changes in the gut microbiota are fatty acids. Excessive fat intake, in general, is a prominent factor inducing obesity. Associations between obesity and MS have already been demonstrated, displaying a positive correlation between body mass index and the risk of developing MS, especially at younger ages [122]. Obesity, defined by the inadequate accumulation of white adipose tissue (WAT), can lead to a state of systemic inflammation called “metaflammation”. Metaflammation occurs, because WAT is not only involved in energy storage, but also functions as an endocrine organ secreting proinflammatory tumor necrosis factor (TNF)-α, IL-6 or leptin. The latter in particular is a cytokine-like hormone profoundly influencing T cell responses in EAE [123–125]. Leptin was shown to enhance phagocytosis and cytokine secretion in macrophages and to promote CD4^+^ T cell proliferation and survival [126], favoring Th1 and Th17 reactions while inhibiting Treg responses [125–127]. In MS patients, monocytes and T cells present in MS lesions and patient-derived cerebrospinal fluid (CSF) both highly express leptin and leptin receptor [128, 129]. However, MS incidence is not necessarily accompanied by weight gain, thus suspecting a direct effect of fatty acids on immunity. Fatty acids, subdivided into saturated and unsaturated fatty acids, were first correlated with MS in the 1950s [130, 131]. More recent work related ω-3 polyunsaturated fatty acids (PUFAs) to anti-inflammatory effects [132]. In contrast, ω-6 PUFAs are precursors of proinflammatory eicosanoids that may promote the activation of the Th17 pathway [133] and are thus suspected to play a detrimental role in a variety of diseases. Studies in the EAE model demonstrated saturated fats to be a risk or beneficial factor depending on their chain length [134]. The increased intake of medium- and long-chain fatty acids (MCFA and LCFA) by the consumption of an experimental ‘Western diet’ was shown to exacerbate autoimmunity in the CNS [134]. This was due to an increased infiltration of Th1 and Th17 cells in the spinal cord. In vitro, the differentiation of murine and human CD4^+^ T cells into Th1 and Th17 cells was significantly increased by the addition of LCFAs. In parallel, the generation of Tregs was suppressed, coinciding with a decreased secretion of anti-inflammatory cytokines. In murine EAE, diets rich in LCFA were shown to modulate the microbiome, such that naïve CD4^+^ T cells are exposed to increased LCFA in the small intestine, thus inducing more proinflammatory T cell responses [134].

Short-chain fatty acids (SCFA) with chain lengths reaching from one to five C atoms mostly occur in the gut as fermentation products of dietary fibers by commensal bacteria [135]. A decrease in SCFA has been observed in patients with IBD [99, 136]. In MS patients, levels of *Clostridia* clusters XIVa and IV were shown to be reduced [137], both formed by diverse bacterial species that are able to produce SCFA such as butyrate [138, 139]. Butyrate displays anti-inflammatory properties, probably indicating that a reduction of these microbes in MS patients may be associated with disease [137–140]. Most data discussing the mechanism for the effect of SCFA demonstrate the involvement of Tregs. In a murine model of IBD, the administration of acetate (C2:0), propionate (C3:0) or butyrate (C4:0) increased the level of Tregs in the gut [136, 141]. In addition, the administration of butyrate to germ-free mice mimicked the effect of *Clostridium* colonization and increased Treg levels in colon lamina propria [142]. Investigating the effects of SCFA in the EAE model also revealed an increase of Tregs, while suppressing the differentiation of Th17 cells [134]. In EAE, feeding propionate ameliorated the disease by promoting Tregs in the small intestine. As a possible mechanism for how propionate might regulate the differentiation of Tregs is the acetylation of histone H3. SCFA, most potently butyrate, were shown to function as histone deacetylase inhibitors, maintaining acetylation of genes important for Treg function, such as *Foxp3* [141–143]. Whether this in vitro effect could also explain the in vivo amelioration of EAE remains unclear. However, synthetic small inhibitors of histone deacetylases have already been shown to decrease inflammation in animal models of arthritis, IBD, asthma, diabetes, cardiovascular diseases, and MS [143]. Thus, SCFA as naturally occurring nutrients [144] or fermentation products may have a possible therapeutic value for autoimmune diseases like MS by potentially triggering the production of anti-inflammatory Tregs.

## Salt

Another typical hallmark of ‘Westernized food’ is the high content of sodium chloride (NaCl). In particular, processed or so-called ‘fast foods’ may contain significantly more NaCl than homemade meals [145]. Recent literature links this high sodium intake to cardiovascular diseases [146], cancer [147], chronic inflammation [148], and also autoimmune diseases [6, 149, 150]. It was demonstrated that primary and secondary lymphoid organs, such as the thymus and lymph nodes, show increased hypertonicity in comparison to the blood [151], indicating that immune cells have to cope with these changes when infiltrating peripheral tissues or during activation in secondary lymphoid organs. Studies dating back in the early 1990s have demonstrated that the activation of innate and adaptive immune cells under hypertonic conditions could enhance immune function, and monocytes were shown to get highly proinflammatory [152], while T cell lines were shown to secrete less anti-inflammatory factors and to produce, for instance, more TNF-α [153]. Moreover, it was recently shown that high salt conditions, mimicking in vivo situations in the tissue after a high salt diet, enhanced the activation of classical M1 macrophages, and increased the expression of proinflammatory mediators [154–156]. In contrast to this, excess salt diminished the activation of the so-called M2 macrophages (M(IL-4 + IL-13)) and decreased their ability to suppress effector T cell proliferation [157]. Similar to cells of the innate immune system, raising the sodium concentrations in vitro also affected adaptive immune cells and promoted the differentiation of murine and human Th17 cells with a pathogenic phenotype, displaying increased expression of, e.g., CSF2 and IL-23R [158, 159]. It was demonstrated that this effect was linked to p38/mitogen-activated protein kinase (MAPK), nuclear factor of activated T cells 5 (NFAT5), and serum- and glucocorticoid-regulated kinase-1 (SGK1) dependent pathways. The fact that in particular pathogenic Th17 cells with a similar phenotype are involved in autoimmune diseases indicates that a high salt diet may represent a previously unrecognized environmental risk factor for autoimmunity. Indeed, in the EAE model, a high salt diet augmented disease onset and severity [158, 159]. Exacerbated disease was accompanied by increased induction of Th17 cells and heightened numbers of CNS infiltrating pathogenic Th17 cells. More recent work focusing on the effects of sodium chloride on regulatory immune cells provided evidence that high salt conditions almost completely block the suppressive function of human and murine Tregs in vitro and in vivo. High amounts of salt induced Th1-like Tregs, secreting IFN-γ in an SGK1-dependent manner [160]. In MS patients, a recently published observational study demonstrated a heightened disease activity and enhanced inflammation in subjects with an increased dietary sodium intake [149]. However, direct human data on effects of high salt intake on the immune system are still sparse. A few available studies indicate a heightened immune activation in association with excess salt intake. In particular, the composition of monocyte subsets was shown to be shifted towards higher numbers of proinflammatory monocytes by a short-term increase in dietary salt intake [161]. Similarly, a recent longitudinal study observed a striking correlation of monocyte numbers and function with salt-intake levels. The consumption of a high salt diet was paralleled by higher monocyte counts and a significant increase in IL-6 and IL-23 production, whereas the secretion of IL-10 was decreased [162]. Intriguingly, IL-6 and IL-23 are major inducers of Th17 cells also in humans. Novel strategies to investigate the body’s sodium content recently showed that a high salt diet is accompanied by a periodical storage of sodium in skin interstitium and the muscle [163–165]. This process represents a new regulatory mechanism independent from water retention and sodium clearance by the kidney. Instead, this extra-renal sodium clearance was shown to involve the immune system. Skin-resident macrophages, activated in an NFAT5-dependent manner, secrete vascular endothelial growth factor c (VEGF-C), a growth factor for lymphatic vessels and, thereby, inhibit the development of salt-mediated hypertension [164]. Initial studies, measuring the sodium content in humans by ^23^Na-MRI, revealed this specific accumulation of sodium in the skin and muscle [163, 166]. The concept that higher interstitial sodium content via increases in dietary salt intake may drive proinflammatory responses of the innate immune system, promoting Th17 cell induction was recently supported by a study of Medzhitov and colleagues. In this study, it was shown that macrophages could sense hypertonic conditions through the caspase-1 pathway, thereby promoting Th17 cell induction by heightened IL-1β secretion [167]. In line with these findings, Jantsch et al. demonstrated that sodium, which accumulated at the site of skin infections, was able to boost proinflammatory macrophage responses in a p38/MAPK and NFAT5-dependent manner [156]. In summary, these data indicate that the sodium balance may affect innate and adaptive immune function and homeostasis at various levels. Salt intake, therefore, might represent an environmental risk factor, which may influence autoimmunity by promoting proinflammatory and blocking anti-inflammatory immune responses. However, it remains to be seen how significant these effects are for human autoimmunity. It is also likely that the genetic architect could play a role here, as recently indicated by an EAE study [168]. Moreover, alterations in the gut microbiota may indirectly contribute to the observed effects of altered immune function and disease [2, 6].

## Conclusion

Increasing research efforts indicate that nutritional factors have the capability to potently modulate autoimmune responses and inflammation. Sodium chloride and saturated fatty acids have been implicated as risk factors in many diseases, such as stroke, hypertension, cardiovascular diseases, chronic inflammation and autoimmunity. It is thus not surprising that there is growing interest in special diets as add-on therapies to conventional therapeutics. Although no long-term clinical trials currently exist, the data discussed above suggest that the impact of components of the daily diet like saturated fats and sodium on inflammatory processes and autoimmunity should be further investigated.

The gut as the main absorption interface for nutritional components displays the most prominent anatomic site that links diet and disease. Recent studies have already suggested a role for the gut microbiome in a number of diseases, including T1D, IBD, and obesity, and present further evidence that the gut microbiome may also play a role in diseases affecting the CNS, such as MS. The impact of gut microbiota and its metabolites on the mucosal immune system was shown to not only affect the gut environment, but also to modulate extra-intestinal immune responses by influencing the balance of pro- and anti-inflammatory T cell subsets. Modifying the gut microbiota, either directly or indirectly through dietary factors, might thus be a potential therapeutic option for the treatment of various diseases, including MS. Promoting the induction of anti-inflammatory Tregs and reducing pathogenic Th17 cell responses might represent here the most promising strategy in the context of autoimmunity.
